# Process-Oriented Feedback through Process Mining for Surgical Procedures in Medical Training: The Ultrasound-Guided Central Venous Catheter Placement Case †

**DOI:** 10.3390/ijerph16111877

**Published:** 2019-05-28

**Authors:** Ricardo Lira, Juan Salas-Morales, Luis Leiva, Rene de la Fuente, Ricardo Fuentes, Alejandro Delfino, Claudia Hurtado Nazal, Marcos Sepúlveda, Michael Arias, Valeria Herskovic, Jorge Munoz-Gama

**Affiliations:** 1 Department of Computer Science, School of Engineering, Pontificia Universidad Católica de Chile, Santiago 7820436, Chile; rlira2@uc.cl (R.L.); jisalas1@uc.cl (J.S.-M.); lileiva@uc.cl (L.L.); vherskov@ing.puc.cl (V.H.); jmun@uc.cl (J.M.-G.); 2 Department of Anesthesiology, School of Medicine, Pontificia Universidad Católica de Chile, Santiago 8331150, Chile; rdelafue@med.puc.cl (R.d.l.F.); rfuente@med.puc.cl (R.F.); aedelfin@med.puc.cl (A.D.); cehurtado@uc.cl (C.H.N.); 3 Department of Business Computer Science, Universidad de Costa Rica, San Ramón 111-4250, Costa Rica; michael.arias_c@ucr.ac.cr

**Keywords:** process mining, healthcare, feedback, medical training, surgical procedures

## Abstract

Developing high levels of competence in the execution of surgical procedures through training is a key factor for obtaining good clinical results in healthcare. To improve the effectiveness of the training, it is advisable to provide feedback to each student tailored to how the student has performed the procedure on each occasion. Current state-of-the-art feedback is based on Checklists and Global Rating Scales, which indicate whether all process steps have been carried out and the quality of each execution step. However, there is a process perspective that is not captured successfully by these instruments, e.g., steps performed, but in an undesired order, group of activities that are repeated an unnecessary number of times, or an excessive transition time between two consecutive steps. In this research, we propose a novel use of process mining techniques to effectively identify desired and undesired process patterns regarding rework, the order in which activities are performed, and time performance, in order to complement the tailored feedback for surgical procedures using a process perspective. The proposed approach was applied to analyze a real case of ultrasound-guided Central Venous Catheter placement training. It was quantitatively and qualitatively validated that the students who participated in the training program perceived the process-oriented feedback they received as favorable for their learning.

## 1. Introduction

The development of procedural skills is critical for physicians of any specialty. Better technical skills are associated with better clinical outcomes [1] and the absence of them is the most important factor in errors derived from the operator in healthcare [2]. Historically, procedural skills have been taught in daily clinical work, in a master-apprentice model, assuming sufficient exposure time to obtain them. However, there are a number of complexities that increasingly make this model more difficult: health system efficiency considerations, time constraints on clinical training activities, and patient safety [3]. In particular, some examples of efficiency issues are mentioned in [3]: resident work hour restrictions have reduced the exposure of residents to their surgical mentors; and changes in reimbursement and other insurance and legal issues have introduced productivity constraints on surgical procedures. Therefore, training in simulation environments prior to contact with patients has expanded as an effective practice to achieve positive effects on the learning process [4]. However, this teaching methodology has a high cost [5] and some of its aspects are not fully resolved, including how to give effective feedback to the students. Critical decisions about feedback are what information to deliver, when it should be given, how it should be delivered, and who should provide it.

Feedback in clinical education of procedural skills is defined as the delivery of specific information on the comparison between the student’s performance and a standard [6]; it ensures that certain standards are met, and promotes learning [7]. Feedback is effective when it is used to promote a positive and desirable development [8]. In the case of procedural skills taught in a simulation environment, feedback can be delivered by an instructor, partner or computer, either during or after the simulation activity [9]. However, standard oral feedback has some drawbacks, since it depends on the availability of a person who is trained in the procedure, who is usually an expensive, difficult to obtain resource. In addition, this type of feedback is essentially the subjective opinion of the evaluator.

To establish when an apprentice has reached an acceptable level of competence that guarantees patient safety, various tools have been developed. The two most commonly used are Checklists and Global Rating Scales (GRS) [10]. Checklists break down the procedure into a series of steps, and check whether the student has performed each step. Meanwhile, GRS consider the evaluation of the student’s performance in different areas. In both cases, at the end of each training session, they allow to provide feedback to the student about which steps/qualitative dimensions deserve a greater attention. Checklists have the limitation of giving similar weights to different errors in the execution of a procedure, even though some of them have greater implications for the clinical outcome and patient safety [11]. On the other hand, GRS provide a more qualitative evaluation, but have the limitation that their reliability is dependent on the characteristics and training of the evaluator [12].

The performance of a surgical procedure can be seen as a process [13], i.e., a set of activities (procedure steps) and events that are executed in a specific order so as to achieve a certain goal. A process-oriented feedback seeks to emphasize the relevance of following this orderly sequence of activities, identifying deviations such as: rework, execution of activities in a different order than desired, slow execution of activities, or slow transition times between activities.

Process Mining [14] is an emerging discipline that allows analyzing the execution of a process based on the knowledge extracted from event logs created from the data stored in information systems. Event logs record the execution of the different activities into which a process can be broken down. The goal of process mining is to discover, monitor and improve real processes. There are three main types of process mining [14]: discovery, conformance and enhancement. Discovery techniques take an event log and produce a process model that describes how the process is actually being performed. Conformance checking techniques take an existing process model and compare it with an event log of the same process; it can be used to check if reality, as recorded in the event log, conforms to the model, and vice versa. Enhancement techniques extend or improve an existing process model by using information about the actual process execution, as recorded in an event log.

Although Process Mining has been commonly used in the context of Business Process Management, it is increasingly common to see its implementation in other areas. Particularly in the area of healthcare, the use of process mining tools has been increasing over time [15], focusing the interest in its clinical applications for evaluation of surgical instruments, optimization of operating room management, development of expert systems, robotic assistance, among others [16].

In this research, we propose a novel use of process mining techniques in order to complement the tailored feedback of surgical procedures using a process perspective. In particular, we believe it is possible to identify when the student repeats some activities (rework), when the student performs some activities in an incorrect order, or when it takes too long to perform an activity or a transition between two consecutive activities. To the best of our knowledge, this is the first application of process mining for providing structured feedback in procedural training.

The proposed method has been validated from the students’ point of view by applying it to a course, taught by the simulation center of the Pontificia Universidad Católica de Chile, where students learn to perform the ultrasound-guided Central Venous Catheter placement procedure. In this course, the traditional method of feedback delivery is immediate oral feedback by the instructor, along with an evaluation based on Global Rating Scales and Checklists.

This article is an extended version of a previous article [17]. In this version, a more detailed description of the case study is provided and further results are presented: a description of the final report provided as feedback to the students, and both a quantitative and a qualitative analysis of the students’ responses to a questionnaire that they answered after the training program.

The structure of the article is as follows: Section 2 describes our research objectives and the training case in which the proposed approach was applied. Section 3 describes the proposed method. Section 4 explains how the training program was intervened so as to validate the proposed approach. Section 5 illustrates how the proposed method can provide feedback to the students. Section 6 presents the results of the validation intervention. Section 7 describes the main limitations of our approach and potential future work. Finally, conclusions are highlighted in Section 8.

## 2. Objectives and Context

This article has three main contributions. First, we propose a novel method for applying process mining techniques to identify desired and undesired process patterns regarding rework, order, and performance, in order to complement the tailored feedback of surgical procedures using a process perspective. Second, we illustrate how this method can be applied to provide feedback to students of ultrasound-guided Central Venous Catheter placement training. Third, we validated, both quantitatively and qualitatively, that the students who participated in the training program perceived the process-oriented feedback as favorable for their learning.

### 2.1. Objectives

Our main research goal is to propose how *process mining* can be used to identify desired/undesired process patterns as part of the tailored feedback on medical procedural training. It can be broken down into specific objectives:

**O1:** To identify desired/undesired process patterns regarding *rework*.
**O2:** To identify desired/undesired process patterns regarding the *order* in which activities are performed.
**O3:** To identify desired/undesired process patterns regarding *performance*.

In addition, we aim at evaluating its impact, by analyzing its use as a feedback tool for students who are learning how to perform the ultrasound-guided central venous catheter placement in a simulation environment.

### 2.2. Ultrasound-Guided Central Venous Catheter Placement Training Case

The Central Venous Catheter (CVC) placement is a frequent [18] and transversal procedure for many medical specialties. CVC is used for drug infusion, monitoring and parenteral nutrition. In some cases, the placement process can have mechanical complications, some of which can be fatal. The use of ultrasound equipment to visualize structures and guide venous puncture is a quality standard of patient care [19,20], with a proven impact on reducing the morbidity associated with this procedure and improving the success rate [21,22]. However, ultrasonography is underutilized [23] and trained doctors do not meet the standards necessary for proper placement [24]. Training in simulation conditions is effective in achieving the necessary skills for an adequate execution of this procedure [11,25,26].

The clinical simulation center at the School of Medicine of the Pontificia Universidad Católica de Chile developed a training program for residents of anesthesiology, emergency medicine, cardiology, intensive medicine and nephrology, in the context of the research “Simulation-based training program with deliberate practice for ultrasound-guided jugular central venous catheter placement” [27].

This program is developed in three stages:

I. Online Instruction and PRE recording: three online classes are available through a web platform, each with mandatory and complementary readings. At the end of this stage, a written evaluation is taken and a recording of a first procedure execution is made (identified as PRE video).
II. Demonstration Session: a demonstration of the entire ultrasound-guided CVC placement in a “Blue Phantom Torso” (http://www.bluephantom.com) is given by an expert to the entire group of residents. In addition, the 4 stations of deliberate practice are presented:
  (a) preparation of the ultrasonography equipment, the patient and the work tools;
  (b) handling ultrasonography equipment;
  (c) venous puncture with ultrasound guidance;
  (d) catheter placement and fixation.
III. Deliberate Practice: residents must complete four deliberate practice sessions accompanied by an instructor who supervises and delivers immediate feedback in the development of stations described in stage II.

In the course, the traditional method of feedback delivery is immediate oral feedback by the instructor during deliberate practices. Each session of deliberate practice is led by an instructor with expertise in the ultrasound-guided CVC placement procedure and in simulation-based education, who provide immediate feedback to students and assure the accomplishment of their learning objectives using a standardized approach and checklists [27]. Students are able to practice as many times as they want during the programmed time per session.

After the end of the course, a second video (identified as POST video) of the ultrasound-guided CVC placement procedure is recorded for each resident, which is used to evaluate the training program.

Parallel to this training session, we recorded videos (identified as EXP videos) of the execution of the same procedure under the same conditions by different professionals from the anesthesiology division, with at least 5 years of clinical practice and experience in ultrasound-guided CVC placement.

## 3. Method

Unlike more classical process mining methodologies, in our approach event logs are not generated automatically from the execution of the procedure. Instead, our method uses an observer-based approach [16], i.e., observation performed by a human observer. In this case, event logs were generated based on the off-line observation, by medical specialists, of the aforementioned recorded videos.

**Figure 1:**
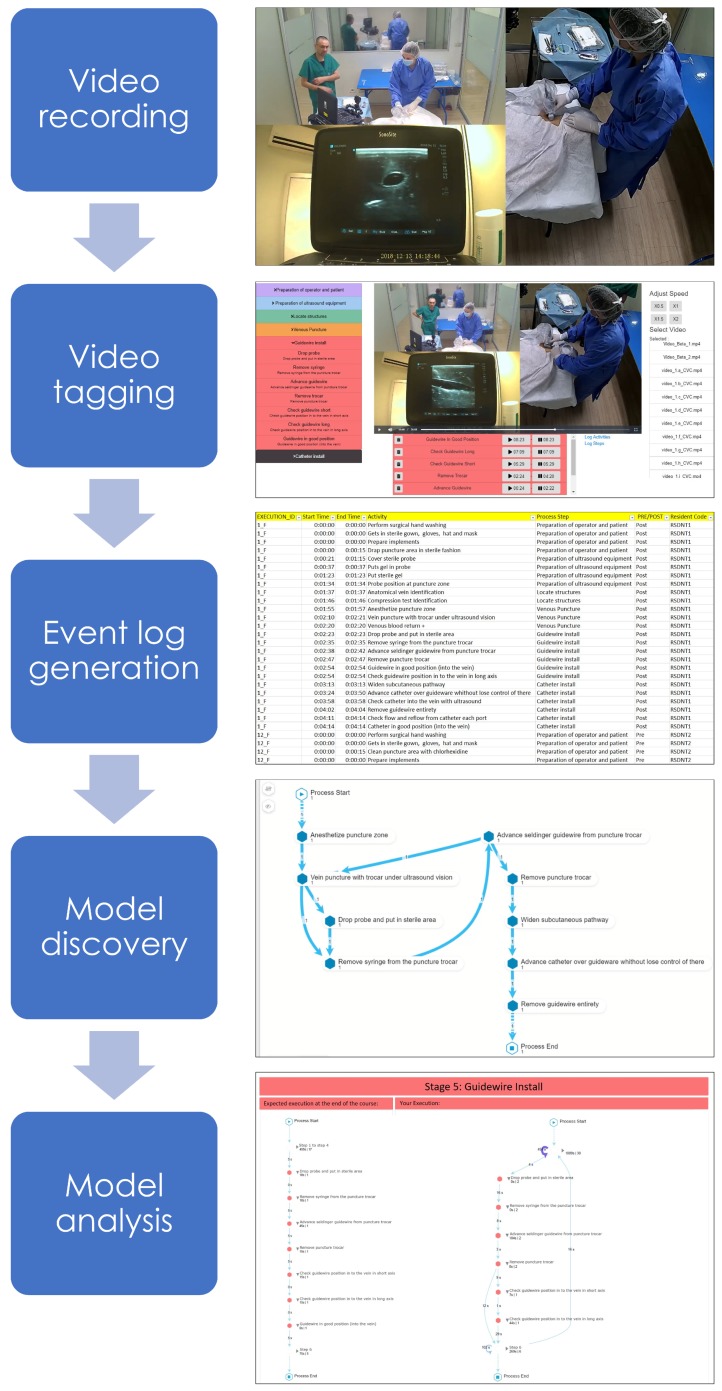
Stages of the proposed method.

The proposed method is inspired by the process mining PM2 methodology [28], which has been previously used in the healthcare domain [15,29] and is suitable for the analysis of both structured and unstructured processes. The proposed method is decomposed into five stages (see Figure 1): (1) video recording—data are extracted from the videos recorded for both students and experts; (2) video tagging—the videos were tagged by two medical doctors, identifying for each case (each execution of the CVC placement), activities (procedure steps) and timestamps (time elapsed since the beginning of the procedure); (3) event log generation—tagging information is used to generate an event log containing the data of all executions; (4) model discovery—process mining discovery algorithms are applied to the event log in order to describe the observed behavior of the procedure; and, (5) model analysis—the discovered process models are analyzed in order to generate feedback for each student.

### 3.1. Video Recording Stage

In this stage, we extracted trace data from different recorded videos that register how the procedure was executed by each of the students. The videos were organized into three categories: PRE: executions previous to the training program, POST: executions after the training program, and EXP: executions by experts.

Three cameras were used for recording the procedure: one recording a general perspective, another focusing on the placement area of the catheter in the phantom, and a last one focused on the support ultrasound. To synchronize the videos various software were tested and the one that was finally chosen was the commercial software “Wondershare Filmora Video Editor” (https://filmora.wondershare.com/video-editor). The result of the synchronization of the videos can be seen in Figure 1.

### 3.2. Video Tagging Stage

In this stage, each video is tagged by two medical doctors. Since video tagging is one of the most important elements to be able to correctly apply the process-oriented feedback, different video tagging software were evaluated, but at the end, we decided to develop our own software, focused on usability, to be able to generate an event log as close to reality as possible, as shown in Figure 1.

**Figure 2:**
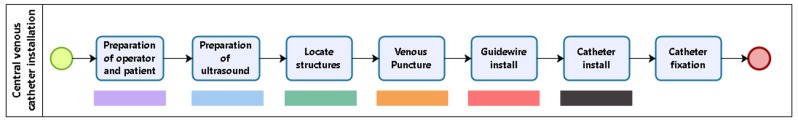
Stages of the ultrasound-guided CVC placement procedure.

The main features of the software are the following:

**Interact with the recorded processes**: The tool allows users to interact with the video in a common way, e.g., play and stop the video, move forward and backwards, mute and allow sound, play the video at different speeds, and display the video in full-screen to observe the details in a clearer way.
**Walking through the list of activities**: A list of activities that are characteristic of the ultrasound- guided CVC placement procedure are used as possible labels for each of the activities observed in the videos. For an easy and more direct access to the activities, they are grouped in sets (with an intuitive name and a color), according to the different stages of the procedure (see Figure 2). Once clicked, a set expands showing the list of activities associated to it. Each activity has a short name (to easily identify the activity), and a longer and more descriptive name to fully understand when the activity should be identified in the video.
**Adding a new event**: When an activity is clicked, it is directly added to the event log, registering the time the video was at the time of clicking as both the start and completion time for the event. Events are displayed in descending order by their starting time to facilitate access for the user.
**Updating start and end time**: Clicking on the start or completion time of an activity will update the time with the video time in the moment of the click. A common action is to create the event at the start of the activity, and wait for it to finish to click on the end time to update it.
**Exporting the event log**: The tool allows exporting the event log to .CSV format, which is supported by most process mining software, and allows adding extra parameters in case it is considered convenient.

Each video of the ultrasound-guided CVC placement procedure contained between 13 to 34 min of recording. The time it took for the medical experts to tag the video was 30% to 50% more than the duration of the video.

### 3.3. Event Log Generation Stage

In this stage, we created the event log that is used by process mining algorithms. The event log is a comma separated value file that included a row for each of the tagged events, grouping in a single file the data gathered from all the videos. It contains the following columns: case id (each video), event (activity name), start and end timestamps (both with a granularity at the second level), and an observation field. In addition, for each video, the following fields are recorded: performer (participant id), type of performer (student or expert), category (PRE, POST or EXP), and success or failure in the execution of the procedure.

### 3.4. Model Discovery Stage

We processed the event log with a discovery algorithm to obtain a process model representing the behavior of each student when performing the procedure. In the PM literature, there is a wide range of discovery algorithms [14]. We selected the Celonis algorithm and its implementation in the Celonis commercial tool (http://www.celonis.com). This algorithm is based on the Fuzzy algorithm concept [30] combined with some characteristics from the family of Heuristic algorithms [14], providing process models that are easy to interpret for an interdisciplinary audience. Moreover, the Celonis tool also integrates a set of filtering options to explore the process data interactively and to address our specific objectives.

### 3.5. Model Analysis Stage

Celonis was used to identify aspects of the execution of the process that can be delivered as feedback to the student to guide their learning. Specifically: (1) identify rework, i.e., when the student repeats one or more activities in the execution of the procedure; (2) identify the execution of activities in an incorrect order of execution, comparing it with the order in which the experts perform it; (3) analyze the student’s performance, including duration of activities and transition time between them, comparing it with the performance of the experts.

We consider two key features to create custom views that can be used to provide process-oriented feedback to the students:

**Filter**: filters can be applied to the event log in order to obtain more specific process models. We use three kind of filters:
  *Activity selection filters:* They allow to exclude some activities from a process model. For example, we distinguished two types of activities: action activities are those that are performed in order to place the CVC, and checking activities are those that are performed in order to verify whether some critical activities produce the desired outcome. In some models, we exclude the checking activities.
  *Case selection filters:* They allow to create a process model using only some process instances. For example, we use these filters to create process models that display only the activities performed by the student we want to give feedback to, either in the PRE or POST scenario, and to create a process model that displays only the activities performed by the group of experts (EXP).
  *Collapsing filters:* They allow to group some activities in a process model, so that they are represented by a single element in the model. For example, some phases of the CVC placement are regularly performed well by most participants. Therefore, all activities corresponding to one of those phases can be clustered in a single cluster element.
**Compose view**: We compose views that include different models of a process based on the data loaded from an event log. Each of those models can be created by applying different filters to the event log in order to obtain more specific process models. In Section 5, some examples of views composed of three models are shown, displaying the execution of the procedure by a student before/after the training, compared to the execution of the procedure by an expert.

## 4. Validation

Kirkpatrick’s four levels of evaluation for training programs [31] are the following: Reaction, Learning, Behavior, and Results (healthcare outcome in the medical education context). In this article, we evaluate whether the proposed process-oriented feedback approach is able to get a favorable reaction (first level) from the students. To measure whether a training activity is valued positively by students, the following instruments are usually used: questionnaires (with open-ended or closed-ended questions), focus groups, or interviews.

The proposed method was validated from the students’ point of view in the ultrasound-guided CVC placement course, through an intervention in which students of the training program were subjected to a formal process-oriented feedback strategy. This intervention was then analyzed through a survey applied to the students once they finished the course. The questionnaire covers both closed-ended questions (to perform a quantitative analysis) and open-ended questions (to perform a qualitative analysis).

The focus of the delivered feedback is two-fold. First, to emphasize the process-oriented approach, for which a process model is presented to the students at the beginning of the course, which describes the order in which the different steps of the ultrasound-guided CVC placement procedure must be executed. And second, to deliver feedback based on this process-oriented approach, through a report that shows the order in which the student performed the activities (being able to detect rework or loops) and the time performance (activities duration, and the time elapsed between them).

### 4.1. Intervention Actions

The feedback is provided through three intervention actions:

(i) *Process-oriented explanation* of the procedure: the procedure is explained to the students through a reference process model, a validated BPMN model that describes the correct way to perform the procedure. The model is presented to the students after the initial online classes and before recording the pre-session video (PRE). Afterwards, both a printed and a digital version of the model were handed over to the students.
(ii) *Video tagging*: students are requested to tag their own video, registering when they perform each step of the procedure. However, the main goal of this intervention action is they can observe their own performance, and detect the mistakes they may have made.
(iii) *Feedback report* delivery: a formal feedback is provided to the students using a feedback report (described in Section 4.3) created so as to compare their performance in the pre-session evaluation (PRE) and the ideal performance, based on the execution of experts (EXP). This report is delivered to the students by an expert in the procedure before the deliberate practices.

### 4.2. Survey

In order to evaluate the students’ perception about the relevance that the process-oriented feedback provided to them has on their learning process, we applied a survey once they finished the course. The objective of the survey is to assess the perception of the students about the impact of the process-oriented feedback in their learning process and to identify improvement opportunities for future implementations. The evaluation survey has 4 sections. The first one is about the general perception about the feedback provided, and then specific sections for each of the 3 intervention actions that make up the process-oriented feedback: “process-oriented explanation”, “video tagging” and “feedback report”.

Participants answered a series of closed-ended questions on a 5-point Likert scale. For example, considering the intervention “Receiving a process-oriented feedback report with an analysis of your own execution”, from 1 to 5, how much did it contribute to your learning? (1: Not at all helpful to 5: Extremely helpful). They also answered a series of open-ended questions about their perception of the usefulness of the different intervention actions.

### 4.3. Feedback Report

A report was generated to provide feedback to the students after having tagged the video recording their first execution of the procedure (PRE), but before continuing with the deliberate practice sessions, so that they could use the feedback obtained when analyzing the video and upon receiving the report, to better focus their practice sessions.

Reports were generated using the Celonis software, mainly because it allows generating a structure of analysis within the same software, downloadable as PDF, which effectively and efficiently provides process-oriented feedback based on the process mining analysis. The report follows the structure of the reference process model of the ultrasound-guided CVC placement procedure, which splits the procedure into 6 sequential main stages, each including specific activities. Figure 2 shows the stages into which the ultrasound-guided CVC placement procedure is decomposed. The report shows a diagram with a general view of the procedure, showing it only at the stage level, and then a specific diagram for each stage. In this way, the information is modularized and the generated diagrams are easier to read. The diagrams consider rework information, repetitions, duration of activities, and the time elapsed between them.

**Figure 3:**
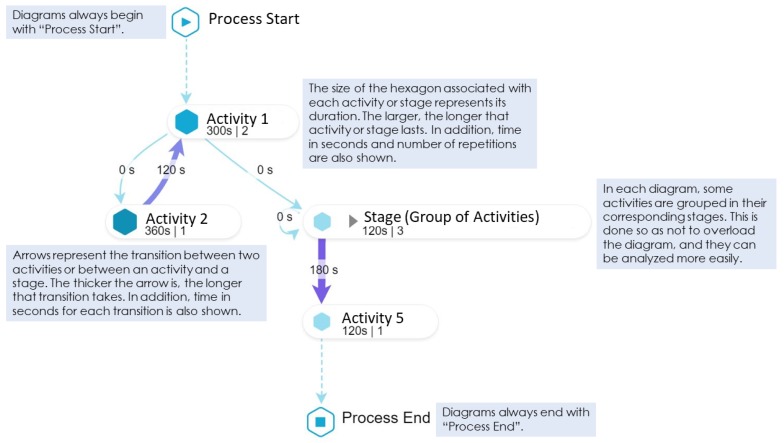
Main elements that compound the diagrams included in the feedback report.

**Figure 4:**
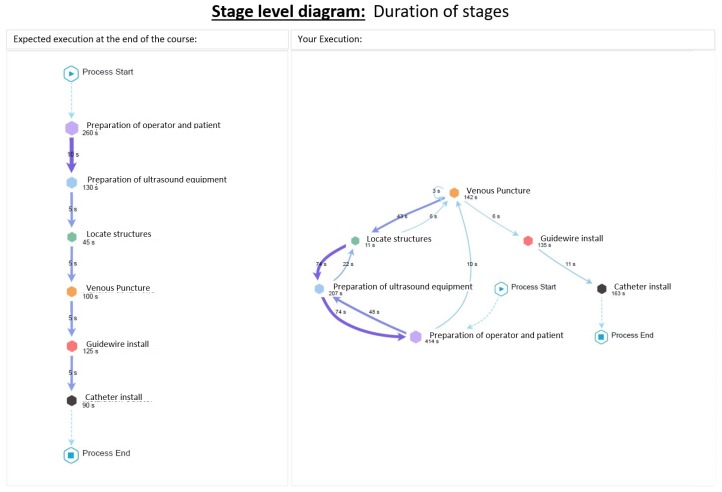
Diagram at the stage level included in the feedback report. The expected execution is shown on the left side (according to experts’ performance) and the student’s performance is shown on the right side.

**Figure 5:**
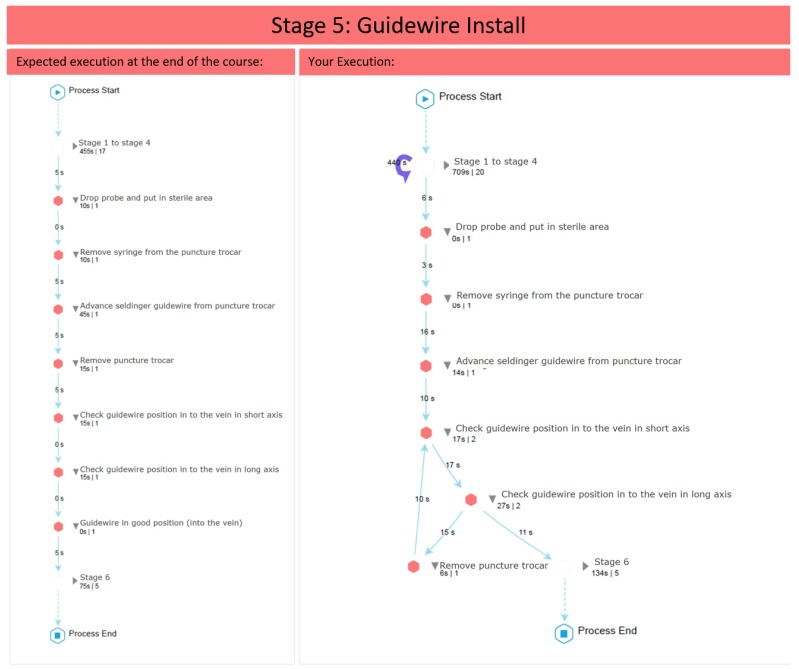
Diagram of the Guidewire Install stage included in the feedback report. The expected execution is shown on the left side (according to experts’ performance) and the student’s performance is shown on the right side.

The report is basically a combination of models, diagrams and brief explanations, as follows. Figure 3 shows the main elements that compound all the diagrams considered. This explanation is provided to the students so as they understand how to read and interpret the diagrams generated by the process mining analysis, since these can be difficult to read for someone who has never seen them, which would hinder a correct delivery of feedback. Figure 4 shows the student’s performance at the stage level, while Figure 5 shows the student’s performance at the Guidewire Install stage (similar diagrams are provided for the other stages).

## 5. Results

The process-oriented feedback for a student who is learning about a surgical procedure is delivered through different process models that show patterns in which their performance is compared to the desired behavior. This information is useful for students because it helps them to focus their attention and effort, so as to avoid making mistakes in future executions. It can be a guide for future sessions of deliberate practice, which allows focusing efforts on simpler and independent tasks, e.g., puncturing the vein with ultrasonography or passing the guidewire, thus simplifying the training to some steps that are difficult due to rework, lack of order, or slowness in execution. Different views and filters were applied to address the three specific objectives:

**O1**. To identify desired/undesired process patterns regarding *rework*.

**Figure 6:**
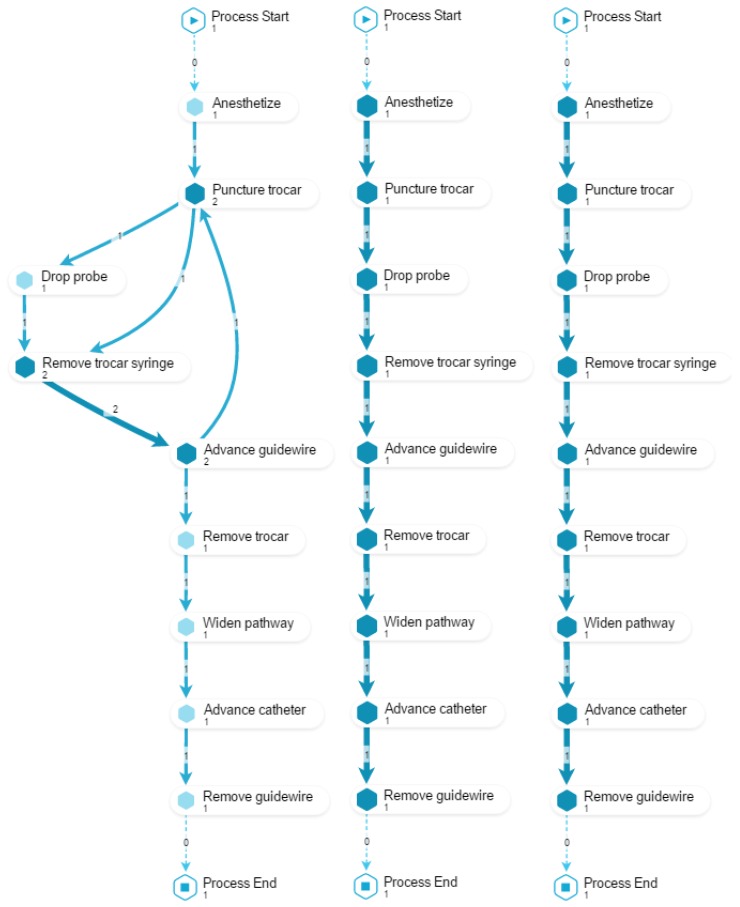
Process model of student *16F69F* in PRE (left) and POST (center), and a generic expert EXP (right). Numbers show activity frequency per case. Activity names are shortened and only action activities are shown for readability reasons. Regarding **O1.1**, the PRE model shows rework during the phase of venous puncture with trocar: the student unsuccessfully performs a first trocar placement, then perform a successful one on the second iteration. Moreover, during the second iteration, the probe is not properly dropped (in the sterile zone) closing the door for a possible third iteration without sterilizing everything again. Notice that, the exact path followed in each iteration can be analyzed using the case animation feature of the tool. Regarding **O1.2**, POST model is able to capture that the same student does not perform any rework, and the process match exactly the reference process performed by the EXP (**O1.3**).

For this objective, we compose a view with three models (as shown in Figure 6). The first two models display how the student performed the procedure before and after the training (PRE and POST). The third model displays how an expert would perform the procedure. Since the purpose is to illustrate the occurrence of reworks in the action activities, checking activities are excluded. This view allows to provide feedback about which activities the student repeated (**O1.1**); to comment on the performance’s evolution, by comparing the reworks observed in PRE versus POST (**O1.2**); finally, by including the execution of an expert, it is possible to compare the student’s performance against the desired outcome (**O1.3**). In the example shown in Figure 6, a rework in the trocar placement can be observed.

**O2**. To identify desired/undesired process patterns regarding the *order* in which activities are performed.

**Figure 7:**
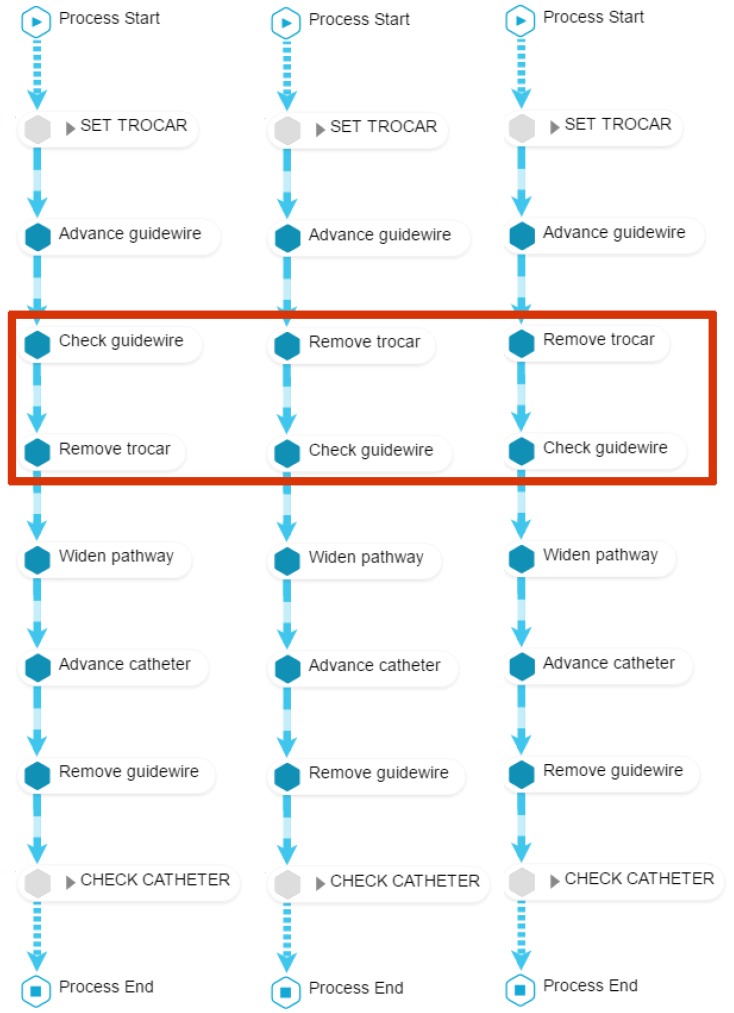
Process model of student *12F67F* in PRE (left) and POST (center), and a generic expert EXP (right). Activities in *set trocar* and *check catheter* phases are clustered for the sake of readability (they show no difference in order between PRE, POST and EXP). Activity names are shortened and both action and checking activities are shown. Regarding **O2.1**, the PRE model shows the student checked the guidewire and then removed the trocar; however, it is desirable to do these activities in the opposite order, because removing the trocar may affect the position of the guidewire. Regarding **O2.2**, POST model is able to capture that the student learned to perform the activities in the right order, and the process match exactly the reference process for the procedure performed by the EXP (**O2.3**).

To achieve this objective, we compose a view with three models (Figure 7). The first two models display how the student performed the procedure before and after the training (PRE and POST). The third model displays how an expert would perform the procedure. Since the purpose is to illustrate problems in the order in which activities are performed, those phases that are correctly performed by most participants are excluded. This view allows to provide feedback about which activities the student performed in the wrong order, or activities that were not performed at all (**O2.1**); to comment on the performance’s evolution, by comparing the performance PRE versus POST (**O2.2**); finally, it is also possible to compare the student’s performance against the desired order as performed by an expert (**O2.3**). In the example shown in Figure 7, *Remove trocar* and *Check guidewire* activities are performed in the opposite order to the desired one.

**O3**. To identify desired/undesired process patterns regarding *performance*.

**Figure 8:**
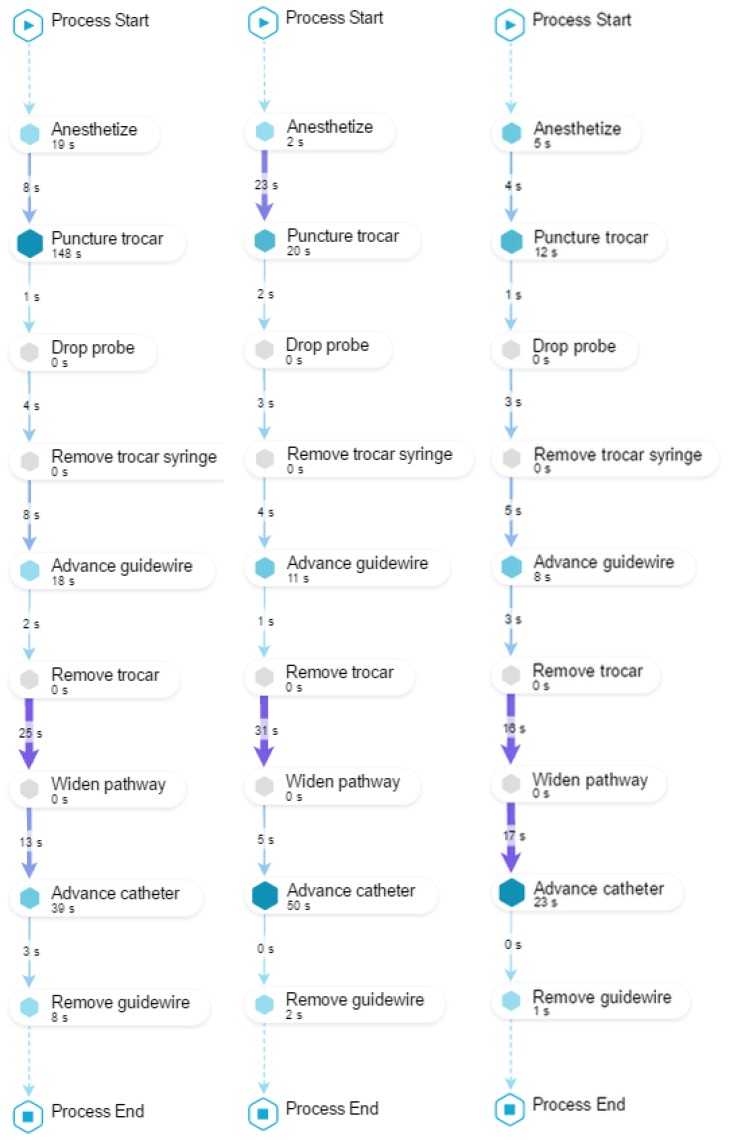
Process model of student *45D59F* in PRE (left) and POST (center), and the average of the 8 experts in EXP (right). Activity names are shortened and both action and checking activities are shown. Regarding **O3.1**, it is possible to highlight *Puncture trocar* was performed too slow in the PRE model (notice the symbol of this activity is larger and darker). Regarding **O3.2**, it can be observed that the transition time between *Remove trocar* and *Widen pathway* is too long (notice the arrow between these activities is wider and darker). Regarding **O3.3**, it is possible to highlight the duration of *Puncture trocar* in the POST model got close to the EXP model. However, the time between *Remove trocar* and *Widen pathway* is still too long (**O3.4**).

To achieve this objective, we compose a view with three models (as shown in Figure 8). The first two models display how the student performed the procedure before and after the training (PRE and POST) including the time required to perform each activity and the time elapsed during the transition between two consecutive activities. The third model displays the average time it takes the group of experts (EXP) to perform the procedure. This view allows to provide feedback about which activities were performed too slowly (**O3.1**), or when the student hesitated taking too much time between activities (**O3.2**). In this case, it is always useful to have as a reference the performance of the experts. In Figure 8, it can be observed in the PRE model that *Puncture trocar* was performed too slowly and that the transition time between *Remove trocar* and *Widen pathway* took too long.

It is also possible to provide feedback about student’s evolution from PRE to POST regarding the duration of activities (**O3.3**) and the time between transitions (**O3.4**). It could happen that some activities (or times between activities) have similar execution times to the average time of the experts, but others are still not close to the average time of the experts. It is then possible to highlight positive aspects, and others where there is still room for improvement. In the example shown in Figure 8, it is possible to highlight in the POST model that the duration of *Puncture trocar* got close to the EXP model, but the transition time between *Remove trocar* and *Widen pathway* is still too long (**O3.4**).

## 6. Validation Results

In total, 12 students answered the survey. The survey allowed us to measure the perception of the students towards process-oriented feedback in general and towards each one of the process-oriented feedback intervention actions, and to assess whether they felt this proposal impacted in their learning process.

### 6.1. Quantitative Analysis

**Figure 9:**
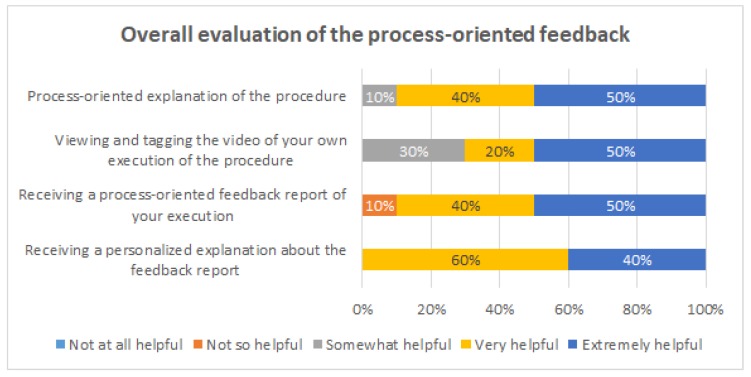
Overall evaluation of the process-oriented feedback.

**Figure 10:**
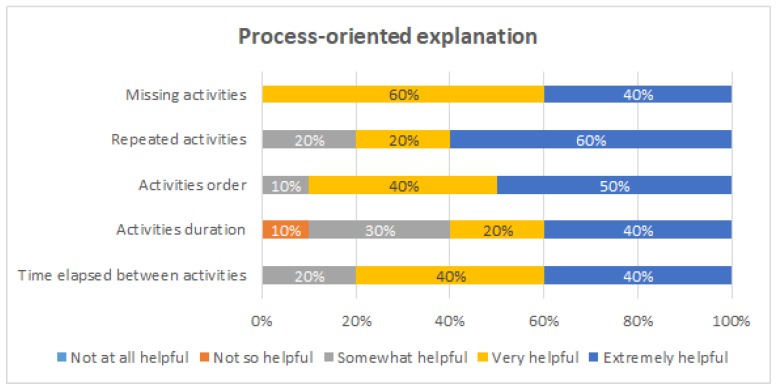
Evaluation of the process-oriented explanation of the procedure.

**Figure 11:**
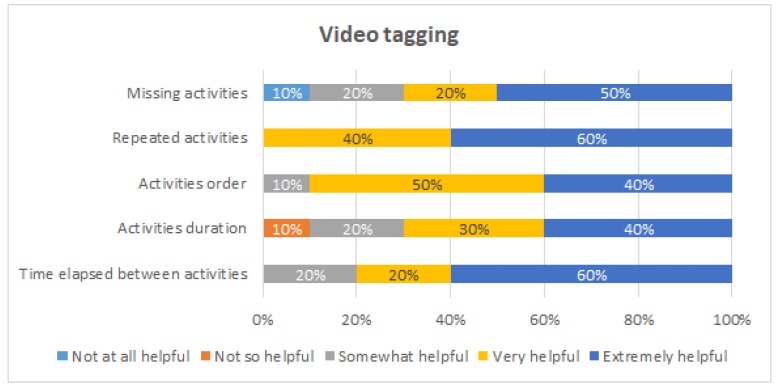
Evaluation of the video tagging.

**Figure 12:**
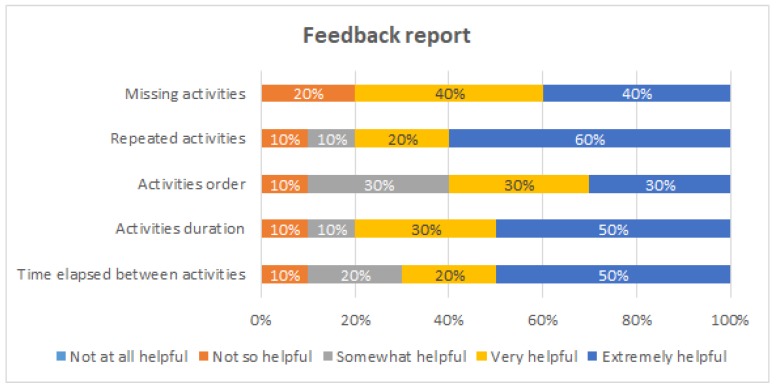
Evaluation of the feedback report.

Figure 9, Figure 10, Figure 11 and Figure 12 show the answers to the quantitative questions of the survey. Figure 9 shows that overall the students assessed positively all the intervention actions. Considering as positive responses the students that answered either “Very helpful” or “Extremely helpful”, the process-oriented explanation of the procedure had 90% of positive responses, the viewing and tagging of their own execution obtained 70%, receiving a process-oriented feedback report of their execution obtained 90%, and receiving a personalized explanation about the feedback report from one of the instructors obtained 100%.

Then, specific questions were asked to evaluate how they felt each intervention action helped them for different learning purposes: to detect missing activities, to detect repeated activities, to be aware of the order of execution of the activities, to be aware of the duration of each activity, and to be aware of the time elapsed between activities.

Figure 10 shows the evaluation of the process-oriented explanation of the procedure. This intervention was considered to be very or extremely helpful for all the evaluated aspects by 80% or more the students, except for being aware of the activities duration, which was still mentioned by 60% of the students.

Figure 11 shows the evaluation of the video tagging. This intervention was considered to be very or extremely helpful for detecting repeated activities by 100% of the students, for being aware of the order of execution of the activities (90%), and for being aware of the time elapsed between the activities (80%). On the other hand, 10% of the students considered it was not so helpful for being aware of the duration of each activity.

Figure 12 shows the evaluation of the process-oriented feedback report. This intervention was considered to be very or extremely helpful by 80% or more of the students for three purposes: to detect missing activities, to detect repeated activities, and to be aware of the duration of each activity. This intervention is the one that obtained the least favorable opinions. At least 10% of the students considered it was not so helpful for each of the evaluated purposes; in particular, 20% of the student considered the feedback report is not so helpful for detecting missing activities.

In summary, all the intervention actions were positively evaluated by the students. At the same time, the results showed each intervention to be more useful for different learning purposes. Therefore, it seems to be convenient to use them all together.

### 6.2. Qualitative Analysis

**Figure 13:**
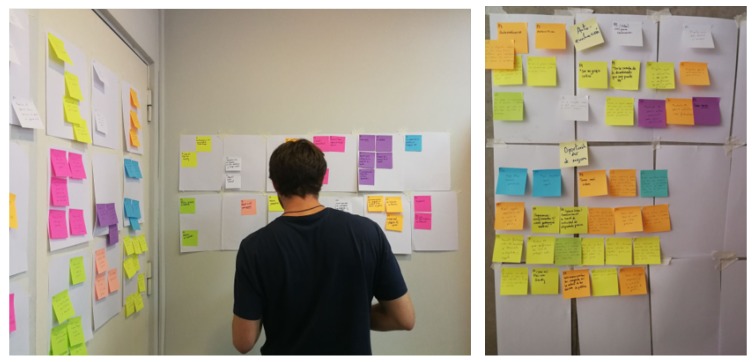
Affinity diagramming process.

The open-ended questions were analyzed with the affinity diagramming technique to evaluate interactive prototypes [32]. Answers to open-ended questions were anonymized and printed out, and an identifier (P1, P2, …, P12) was assigned to each participating student. Three researchers independently read the answers and created post-it notes. Then, a few days later, the researchers met in a dedicated room and iteratively created categories and subcategories, while discussing where to place post-it notes (shown in Figure 13). The entire process lasted about 5 h. Data were analyzed in Spanish (the native language of both participants and researchers).

The affinity diagramming process resulted in four main categories: course feedback methodology, understanding and improving the medical procedure, intervention improvements, and self-awareness, each with several subcategories. Categories and subcategories (in italics) are described below. Quotes from participants (identified by P1–P12) were translated to English and are provided to illustrate some of the concepts. 

**Course feedback methodology**

Participants highlighted *positive aspects of the intervention*, saying, e.g., “This type of analysis is a very good complement to deliberate practice” (P9), and that it was “well-conceived and well carried out” (P11). Two participants were so positive about the intervention that they suggested *expanding the initiative* to other courses: “Every procedure should be taught with process-oriented feedback. It allows improvement of techniques and being aware of fundamental steps in procedure execution.” (P10). A few participants mentioned *not having enough time* to dedicate to the activity, e.g., that the available time slots for the activity were limited and that their numerous responsibilities prevented them from fully engaging in the activity. Participants also offered *suggestions for improvement*, e.g., “a previous tagging activity could be interesting, to help users become familiar with the activity” (P1), “it would be useful if an email had been sent with explanations, before the tagging activity” (P7), and that it would be better to have at least two video recordings per user. 

**Understanding and improving the medical procedure**

Several participants’ comments relate to how the intervention allowed them to *understand* the procedure in a better way. The activity “provides a real and objective view of the procedure, which gives us a good foundation from which to work on” (P11). Participants especially highlighted how they felt an improvement in their understanding of aspects related to the procedure *order*, based both on the video tagging and the feedback report: “It is very useful to realize the order in which you did things, even if you studied the logical initial order” (P2), and *time*: “(The video) is useful to control your perception of time regarding the activities you must do” (P12). Two participants commented that they felt process-oriented feedback would allow them to not only understand the time needed for each part of the procedure, but to optimize it as well.

Participants also commented on how the activity would allow them to *improve* how the procedure is carried out: “decreasing errors” (P9), “systematizing the process” (P4), “improving results regarding process execution” (P7), “not forgetting again what you forgot the first time or what you didn’t initially know” (P5). Another aspect of this improvement is that the activity would allow them to *detect problems*, e.g., allowing them to “become aware of reiterative activities” (P10), and that the feedback “on errors I made is very useful to work on those aspects I need to improve” (P12). 

**Intervention improvements**

A few users had comments on how to improve the *usability* of the application: “It was good to watch and tag the video, but the software could have been better” (P8), “Most are not used to these platforms and focusing on knowing how to use the program distracts from the tagging itself. If it were more user friendly, it would be better” (P1). There was some *difficulty in understanding* parts of the activity: the feedback report was found to be “messy” (P4) and “difficult to understand at first” (P2), while another participant stated “at first, I did not clearly understand how and why to tag the video” (P7), and another said that “getting used to the nomenclature takes time” (P1). 

**Self-awareness**

The final category is related to how participants felt about their own experience and how they learned during the activity. Participants felt they could be *self-critical*: “It was a good moment to evaluate and criticize myself regarding this procedure” (P3), “By seeing myself do the procedure, I could become my own critic and realize that there were steps of the procedure that I didn’t do in the best way” (P9). They stated that this helped them achieve *learning*: “I felt that the deliberate practices were much more useful because I knew what I had gotten wrong or had been difficult during the first recording” (P5), “Getting feedback is very good to help put processes in order in my mind before I carry out a procedure” (P5).

## 7. Limitations and Future Work

The main limitation of this approach is its scalability when a large number of videos needs to be tagged. However, in specialized medical procedure training, as ultrasound-guided CVC placement, it is a viable option. Another potential limitation is when the medical procedure has more complex patterns, e.g., when the order among some activities is not relevant. In such a case, advanced conformance checking techniques should be considered.

It should be noted that in the video tagging, only activities and events that explicitly appear in the reference process model were considered. However, from the medical training point of view, it would also be interesting to incorporate other events: activities that undo previous steps, e.g., remove the catheter; unexpected activities, e.g., check the position of the guidewire with the needle still on; or activities that reflect hesitation or unnecessary movements. Incorporating these events represents an interesting line of future research. For this purpose, a systematic observation of the videos by medical experts is required, in order to establish a classification of the events not considered in the reference model, so they can be considered in the video tagging.

In this article, we evaluate whether the proposed process-oriented feedback approach is able to get a favorable reaction (first level of Kirkpatrick’s four levels of training evaluation [31]) from the students. As future work, we plan to evaluate whether the proposed approach is able to produce an impact on how much the students learn (second level) and compare it with the traditional feedback approach. To this end, the feedback methodology proposed in this article will be applied to a group of students, the experimental group, while at the same time, only the traditional feedback employed in the ultrasound-guided CVC placement training case will be applied to a comparable group of students, the control group. To measure whether the process-oriented feedback has a statistically significant impact on students’ learning, the performance of the two groups on the PRE and POST scenarios will be evaluated using GRS and Checklists, so as to compare whether the improvement of the experimental group in these metrics is larger than the improvement of the control group.

In Process Mining, conformance checking techniques are those that allow comparing how a process is actually being performed, based on the data recorded in an event log, to an ideal reference, as defined in an existing process model. Fitness and precision are the two most common metrics that describe the differences found between the model and the event log. Fitness measures whether the existing model allows for the behavior seen in the event log. Precision measures whether the existing model does not allow for behavior completely unrelated to what was observed in the event log. These two metrics could also be used to measure the students’ performance on the PRE and POST scenarios, and therefore to measure the difference in learning achieved by the experimental group versus the control group.

The automation of the video tagging process is also a future research challenge. Preliminary results using deep learning algorithms show it is possible to recognize some objects and to track the student movements. However, it is challenging to identify specific activities. Another approach is to research whether the video tagging performed by the students themselves is suitable for generating the process-oriented feedback report. As future work, we are considering studying the differences between the event logs obtained from the video tagging performed by students and the one performed by medical experts. We expect there will be some differences: it may be more difficult for students to recognize certain activities or events, or to determine homogeneously when activities start or end. However, if we understand what the differences are, we could conduct an initial training with the students so that they learn to perform the video tagging correctly. If students were able to reliably tag their own videos, it could significantly increase the usefulness of the approach proposed in this article, since students could record each of their deliberate practices, tag their videos, and then obtain and analyze the process-oriented feedback report without requiring the participation of any medical expert.

## 8. Conclusions

In this article, it has been shown that process mining can be used to provide a process-oriented feedback to students who are learning procedural skills, by identifying desired and undesired process patterns regarding rework, order, and performance, in order to complement the tailored feedback of surgical procedures. The approach has been effectively applied to analyze a real ultrasound-guided CVC placement training case. It was quantitatively and qualitatively validated that the students who participated in the training program perceived that the process-oriented feedback could have an impact on their learning. The result of the applied survey shows that the proposed process-oriented feedback is highly valued by students because it allows them to have a better understanding of the procedure and increase their self-perception regarding their performance, especially in aspects of order, rework and missing activities, which are required for a correct execution.

To the best of our knowledge, this is the first application of process mining for providing structured feedback in medical procedural training. It opens a novel approach to the analysis of training programs by generating tailored feedback to students. This approach is generic and therefore can be replicated in other medical training programs.

## Abbreviations

The following abbreviations are used in this manuscript:

BPMN	Business Process Model and Notation
CVC	Central Venous Catheter
PODS	Process-Oriented Data Science
PODS4H	Process-Oriented Data Science for Healthcare
